# Health conditions and occupational risks in a novel group: waste pickers in the largest open garbage dump in Latin America

**DOI:** 10.1186/s12889-019-6879-x

**Published:** 2019-05-16

**Authors:** Vanessa Resende Nogueira Cruvinel, Carla Pintas Marques, Vanessa Cardoso, Maria Rita Carvalho Garbi Novaes, Wildo Navegantes Araújo, Antonia Angulo-Tuesta, Patrícia Maria Fonseca Escalda, Dayani Galato, Petruza Brito, Everton Nunes da Silva

**Affiliations:** 1 University of Brasília, Faculty of Ceilandia, Brasília, Brazil; 2 School of Health Sciences and State Secretariat of Health of the Federal District, Brasília, Brazil

**Keywords:** Epidemiology, Occupational health, Solid waste segregators, Social conditions, Accidents, Medical waste disposal, Public health, Environmental pollution

## Abstract

**Background:**

The inadequate management of solid waste impacts populations’ health and quality of life, and disproportionately affects developing countries. This study aims to describe a protocol for epidemiological diagnosis, the purpose being to estimate the prevalence of chronic and communicable and non communicable diseases in waste pickers, and the occupational and environmental risk factors to which these are exposed.

**Methods:**

This is a cross-sectional study, based on survey design in an area of extreme social vulnerability – the largest garbage dump in Latin America. Using a multidimensional research protocol, divided in three stages*:* 1- The identification of the subjects, and the scheduling of tests; 2- Situational diagnosis through interviews, anthropometric evaluation, measuring blood pressure, collecting hair and nail samples to detect exposure to heavy metals and undertaking laboratory tests; 3- The return of the waste pickers to receive the test results, followed by referral to the health team and to report occupational accidents.

**Results:**

One thousand twenty-five waste pickers undertook tests and interviews. The majority were women (67.0%), with 36–45 years old (45.7%), and 96.0% had children. In total, 27.3% of the participants did not attend to any school and 47.7% were educated only up to primary level. The majority of waste pickers (68.70%) reported accidents and most of them (89.69%) were related to sharp objects. The mean time working in this open dump was 15 years. According the anthropometric measure, 32.6% were overweight and 21.1% were obese. The most common reported diseases were: osteomuscular disorders (78.7%); arboviruses (28.6%); episodic diarrhea (24.9%); hypertension (24.2%); bronchitis (14.3%); intestinal worms (12.6%) and diabetes (10.1%). According to the blood tests, the values outside the reference limits were: Uric acid (23.89%); creatinine (54.06%); GGT range (16.04%); SGOT - Serum Glutamic Oxaloacetic Transaminase (5.29%); SGPT - serum Glutamic-Pyruvic Transaminase (35.52%).

**Conclusions:**

This study is the first to evaluate multiple risks and diseases in the majority of waste pickers working in the largest garbage dump of a continent. These findings highlight the importance to address urgently the environmental, social and health impacts related to the management of solid waste in developmental countries to protect these workers and their families.

**Electronic supplementary material:**

The online version of this article (10.1186/s12889-019-6879-x) contains supplementary material, which is available to authorized users.

## Background

Managing solid waste is the main challenge facing cities in developing countries. It has been calculated that approximately 1.3 billion tons of solid waste were generated in the world’s cities in 2012 – a number which could rise to 2.2 billion tons in 2025 [1]. In Brazil, the increasing consumption of goods has generated a huge volume of waste which achieved 214.868 tons per day in 2017, and 1035 kg per capita per day with an annual growth of 1% [2]. The inadequate management of solid wastes has major environmental and social impacts, and places at risk the health and quality of life of urban populations. This problem is more serious in the developing countries, where less than 30% of domestic garbage is treated appropriately. Furthermore, there is large-scale involvement of informal workers in the collection and sale of these solid wastes – notably in low and middle income countries [3]. It is estimated that, worldwide, some two million individuals work informally as waste pickers. These people are the first to suffer the consequences of the inadequate management of solid wastes [1]. The intensity and type of risks to which these waste pickers are exposed depend on where they work (recycling centers, warehouses, on the streets or in garbage dumps), on their working conditions (informal or organized groups), on the nature of the waste (composition, components and decomposition), and on the duration of their exposure [1, 4–6].

Studies have shown that waste pickers experience situations which place them at high risk of developing morbidities – mainly external and internal injuries e.g., being caught in processing equipment; being run over by trucks; fires; explosions; being injured by glass, contaminated needles, medical waste and also death. They can also develop respiratory diseases, eye infections, stomach problems, typhoid fever, diarrhea, musculoskeletal disorders and carcinogenic effects [7–9]. Individual risk factors, such as poor hygiene practices and lack of access to personal protection equipment or the inadequate use of the same, as well as living conditions which are associated with extreme social vulnerability and inhumane conditions, associated with the use of intoxicating substances or stimulants affecting the central nervous system, can further exacerbate their health vulnerabilities [7]. It’s is common to find a high prevalence of falls, accidents, waterborne diseases and dermatological problems among these workers – as well as high incidence of infections of the reproductive and urinary systems among the female workers, probably caused by the lack of sanitary installations and potable water in the areas where the waste is collected and subsequently treated, as well as by the inadequate treatment of the waste [8].

One study undertaken in South Sudan, Africa, evidences that waste pickers working with recyclable materials are exposed to cuts, maiming, fatal accidents, contamination by heavy metals and dangerous wastes and are at risk of developing pulmonary diseases, HIV and hepatitis C as a result of contact with sharp items and hazardous health waste [10]. A study conducted in India with waste pickers who worked in three different cities reinforced these occupational health hazards highlighting the lack of provisions of protective equipment, low income of the informal workers, along with the ignorance of the workers as aggravating risk factors to different types of external injuries. The major occupational health issues reported by various categories of waste workers were muscle and ligament sprain, cuts and lacerations and different allergies [11]. According to the South Sudanese Development Organization (SSDO), the risks to the health and safety of waste pickers around the world in order of prevalence rate are: joint pain; injuries / cuts; respiratory problems; gastrointestinal disorders; fatigue; skin infection; infectious diseases [10].

One systematic review analyzed the association of exposure to toxic and industrial wastes (hazardous waste) with events related to illnesses and health disorders [12]. From the 57 studies included, the authors showed sufficient evidence to associate exposure to waste from the petroleum industry with various types of acute symptoms, such as neurological, otorhinolaryngological, respiratory, digestive and dermatological. The evidence is limited, however, there are information in relation to cancer of the liver, bladder, breasts and testicles; non-Hodgkin lymphoma; asthma; congenital anomalies in general; anomalies of the neural tube and urogenital, connective and musculoskeletal systems; low birth weight; and preterm birth. The methodological limitations of the primary studies included in the systematic review were indicated as a weak point, given that most of the studies had ecological designs [12]. A separate study conducted by the World Health Organization also pointed out methodological limitations of studies researching the effects on health of exposure to solid wastes [13].

Most studies investigated exposure to waste in the general population. Waste pickers working with solid waste, however, are more vulnerable, as they spend a large proportion of their time exposed to the risks [1]. In addition to this, significant failure to comply with public health legislation on the discarding of health waste from the health services and from people’s homes further increases these workers’ exposure to the risks [14]. It should be emphasized that these workers experience appalling living conditions, are characterized by low educational levels, have low incomes and receive limited support from their governments, particularly in Asia, Africa and South America [15]. As a result, for public health researchers to determine the impact of this work on these individuals’ health, to establish causal relations and to exclude other causes is a major challenge, given that other exposure to other factors in this environment could potentially cause the same results. Furthermore, some clinical outcomes – such as cancer and other forms of degenerative disorders – are manifested months or even years after the initial exposure [16].

One study that assessed 53 publications related to the health conditions of waste pickers who worked in Latin America on the streets, in warehouses belonging to associations and in open-air garbage dumps found that 75% of the studies were undertaken in Brazil, 13.2% in Colombia, 5.7% in Mexico, and 1.9% in Argentina, although two studies (3.8%) discussed simultaneously the work of waste pickers in Brazil, Colombia, and Mexico. The following health issues were raised: irritability, pains in the body and joints, tiredness, coughing, shortness of breath, asthma, insomnia, burning in the eyes, itching, nausea, weight loss, anemia, abdominal pains, discoloration on the skin, allergies, dengue fever, helminthiasis, sexually transmitted diseases, hepatitis, tuberculosis, cholera and mental health problems [17]. A systematic review was conducted to assess consequential health conditions and occupational risks that affect waste collectors in Brazil. The results showed that, as expected, the sites are full of occupational hazards to the workers presenting long working hours; exposures to physical, chemical, mechanical, biological, ergonomic and social agents; and frequent work accidents resulting in psychological illnesses [18].

Many studies have been published on the possible effects on the health of populations who live in the vicinity of landfills, incinerators and garbage dumps. However, our analysis indicates the weakness of the results of the studies available, due to problems of methodological design – mainly related to the lack of information on exposure, and the failure to control for possible confounding factors such as failure to monitor the health, working and living conditions of the populations investigated. Consequently, there is considerable controversy about the possible health effects associated with different wastes. Worldwide, two million informal recyclers work in this business, particularly in the low and middle income countries, where waste management is undertaken poorly, causing significant impacts on health, the environment and the economy [1]. The management of solid wastes, in the developing countries, has received less attention from politicians, researchers and academics than other urban environmental problems, such as air pollution and the treatment of water [19]. According to the sustainable development goals (SDGs), the relevance of this topic of protection of the environment and preservation of health through the management of solid wastes has been increasingly emphasized [20]. It follows that the management of solid wastes and the associated environmental, social and health impacts must be priorities for decision-makers and researchers, so that they can offer solutions for preserving and protecting health and cities’ environments – in particular the health and environments of those working with solid wastes [3, 19, 21].

At the present time, Brazil has nearly 3000 unregistered dumps or landfills, impacting the quality of life of 77 million Brazilians. Approximately 29.6 million tons of waste were placed in controlled dumps and landfills in 2016 [22]. In 2010, Law 12,305 was approved, instituting the National Solid Waste Policy (*Política Nacional de Resíduos Sólidos* - PNRS); this stipulated the eradication of the dumps by 2014. The PNRS, furthermore, calls for selective collection, understood as the recovery of solid waste separated by constitution or composition, the municipalities being responsible for implementing this by contracting the associations and cooperatives of waste pickers to separate and sell the waste [23].

The Brazilian model for managing solid waste takes into account the need for the social inclusion of the waste pickers and the formalization of their role; they need to work safely and legally, using equipment which is compatible with technical, environmental and public health regulations. As a result, these workers need to be valued and supported as strategic allies for the legislation to lead to practical gains, and for companies and the authorities to achieve their goals of recycling packaging within the concept of “shared responsibility”. The Brazilian experience of integrating the waste pickers into the productive recycling chain as principal environmental agents has been reproduced by CEMPRE (Business Commitment to Recycling – *Compromisso Empresarial para a Reciclagem*) in various countries of Latin America. In Brazil, in 2013, the Institute of Applied Economic Research (*Instituto de Pesquisa Econômica Aplicada*) identified 387,910 workers as waste pickers [24].

Brazil is divided into three levels of autonomous governments: municipalities (counties), which act at the local level; states, which are the intermediate subnational level; and the Union, which represents the national government. All of these levels of government finance the Unified Health System (*Sistema Único de Saúde*– SUS, Brazil), the public health system in Brazil. Created by the Federal Constitution of 1988, the SUS ensures the right of all Brazilians to health care. Governed by the principles of decentralization and social participation, the SUS is a single-payer system that provides universal, egalitarian and comprehensive health care aimed at meeting the health needs of the population [25, 26]. In this respect, the primary community-based intervention is the Family Health Strategy (FHS), which delivers a set of individual and collective health actions, including health promotion and protection, disease prevention, diagnosis, treatment, rehabilitation, harm reduction and health maintenance [27]. FHS teams are composed of, at the minimum, one physician, one nurse, one nursing assistant and four to six full-time community health agents. Since its introduction at the national level in 1994, the FHS has expanded fairly rapidly, particularly in the 2000s, reaching 64% of the population (127 million people) in 2014 [28].

This study aims to describe the protocol elaborated for situational diagnosis of health conditions and vulnerabilities of waste pickers, in order to assess the occupational risks and socio-environmental determinants affecting individuals working as waste pickers, managing solid waste in an extremely socially vulnerable area of Brazil. Depending on the participants’ results, they were referred to their Family Health Team for appropriate treatment.

## Methods

The aims of the Epidemiological **S**tudy are as follows: 1. To estimate the prevalence of chronic and communicable and non-communicable diseases in waste pickers, and the occupational and environmental risk factors to which these are exposed**;** 2. To characterize the waste pickers according to sex, age, race, educational level and number of children; 3. To identify the participants’ socioeconomic status (income, educational level, work conditions, marital status, housing conditions); 4. To describe lifestyle (sexual behavior, use of illicit substances, leisure and physical activities) and nutritional status (type of food consumed); 5. To correlate their health conditions with the occupational and environmental risk factors to which they are exposed; 6. To report occupational accidents; 7. To refer them to be followed by the local Family Health Team for appropriate treatment; and 8. To carry out educational actions relating to health and the environment with the waste pickers.

### Study design

This study proposes a protocol for conducting a survey study of the waste pickers employed in an area of extreme socioeconomic vulnerability – the Estrutural Open Garbage Dump (Lixão da Estrutural) located in the Federal District of Brazil. The protocol is divided in three stages. The first relates to the identification of the subjects and the scheduling of tests. The second relates to the situational diagnosis and involves four parts: (i) Reception, individual explanation about the study and the signing of the consent document; (ii) Laboratory analyses and examinations(blood tests and the taking of hair and nail samples to test for exposure to metals (iii) Anthropometric measurements and the measuring of cardiac frequency and blood pressure; and (iv) An interview designed to gather information about demographic status, socioeconomic status, lifestyle, nutrition, health conditions (diabetes, hypertension, cancer, kidney diseases, dermatological diseases, respiratory diseases, allergies, waterborne diseases and mental disorders) and access to the health services (medicines, tests, primary and secondary care. The third stage consisted of the return of the waste pickers to receive their test results, followed by their referral where necessary to the Family Health Strategy team, either for treatment or to report occupational accidents.

### Study locale

**Fig. 1:**
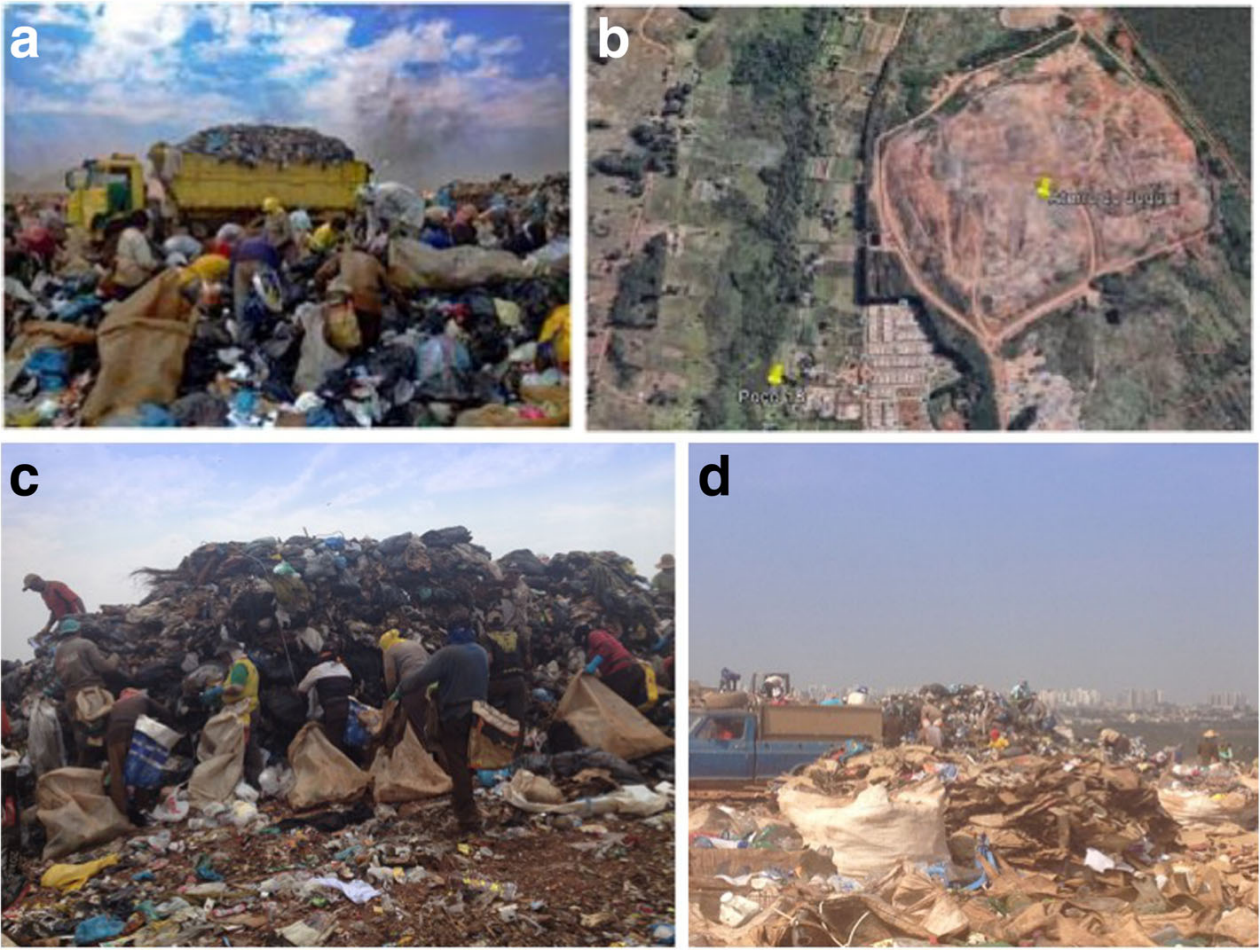
Study Locale. Dumping from the trucks (**a**). An aerial photograph of the garbage dump (**b**). The process of selecting garbage in bags by the waste pickers and the height of the accumulated garbage (**c**) and the closeness of the city (**d**)

The first stages of the protocol are being applied in Brasília. The open-air landfill site there is the largest in Latin America – and the second-largest in the world, after that of Jakarta, in Indonesia – and is considered the most unsanitary in Brazil [29]. First opened in the 1960s, the Estrutural Garbage Dump covers an area of 201 ha, equivalent to 280 soccer fields, and receives the solid waste produced in the Federal District – totaling 40 million tons of waste over the period of its existence. It is located 15 km from the Center of Brasília (Fig. 1), close to the Brazilian National Park (a conservation area) and the Cabeceira do Valo river, along which smallholders produce vegetables and fruits. Around the Estrutural Garbage Dump, there is a large area of environmental degradation - a center of social conflict caused by the construction there of precarious housing inhabited by the waste pickers involved in recycling materials and by people with no other residence. The Federal District produces 1.0 kg (equivalent 2.20 lb) per capita per day, around 8500 tons of garbage per day, of which 2500 tons are solid urban waste and over 6000 tons result from civil construction [29]. Of the garbage produced by homes and business, only 300 tons (12%) is recycled, constituting the raw material for the waste pickers working with solid waste, in the cooperatives and associations or autonomously [29].

Currently, the city of Estrutural (a city which arose due to the settlement of individuals working in the handling or collection of solid wastes) has a population estimated at 35,801 inhabitants, and has the lowest Human Development Index in Brasília, Federal District [30]. At the time of writing, the city has 10 family health teams and just one Primary Healthcare Center for attending to the health of the population.

Brasília only succeeded in achieving the goal of eradicating the largest garbage dump in Latin America in January 2018 (Fig. 1). Among the actions in the Government’s intervention plan for closing down the activities there, which began in 2015, were: the prohibition of accepting out of date foods; the building of a fence around the entire perimeter; identification at the entrance; the installation of chemical toilets; the draining of slurry and burning off of gas; and the inauguration of a landfill site. Since then, the waste pickers have been allocated to what are termed Selective Collection Centers, where waste recycling is undertaken on a safer and more organized footing. Before they commence employment, they are included in this study so that a baseline of their health conditions may be obtained (situational diagnosis).

### Study population

All the workers recorded in the non-electronic information system compiled by the Urban Cleaning Service (SLU), with more than 6 months of activity as waste pickers in the open garbage dump are being included in the study. Waste pickers were registered on this system in the year 2017 so that after the closure of the Estrutural Garbage Dump it would be possible to allocate them to selective collection centers. According to studies already undertaken, the number of people involved in the activity of collecting waste fluctuates according to factors such as the offering of employment and the income obtained through recycling [1]. According to the Urban Cleaning Service, there were about 1200 waste pickers registered in the cooperatives linked to the materials recovery facility before the garbage dump was closed and whose main source of income was from their involvement in the recycling process. To ensure the viability of the protocol, the first stages are being implemented in the Federal District, Brasilia, Brazil. More than 1000 waste pickers have been contacted and have indicated their interest in participating in the study.

### Procedures

#### Stage 1—identification of the subjects and scheduling tests

In order to invite the waste pickers to participate in the study, certain strategies were adopted: (1) Individual invitation based on key informants (presidents of the cooperatives) so that service users could be interviewed and biological samples collected in order to undertake the tests; (2) Invitation by telephone contact and (3) Active search during training sessions of the same to enter the materials recovery facilities and the garbage dump itself. There were no monetary incentives for the waste pickers to participate in the study. They were convinced to participate by the explanation of the importance to know about their health conditions and the opportunity to receive free treatment according to their needs.

These actions were arranged in partnership with the Urban Cleaning Service, the Federal District local government and the Public Health System. The first phase consisted of contacting the waste pickers linked to the Family Health Strategy teams with Community Health Workers, while the second focused on contacting waste pickers who were not covered by the Family Health Strategy. The aim was to ensure that the health needs of all involved in waste picking were met.

#### Stage 2- situational diagnosis

**Fig. 2:**
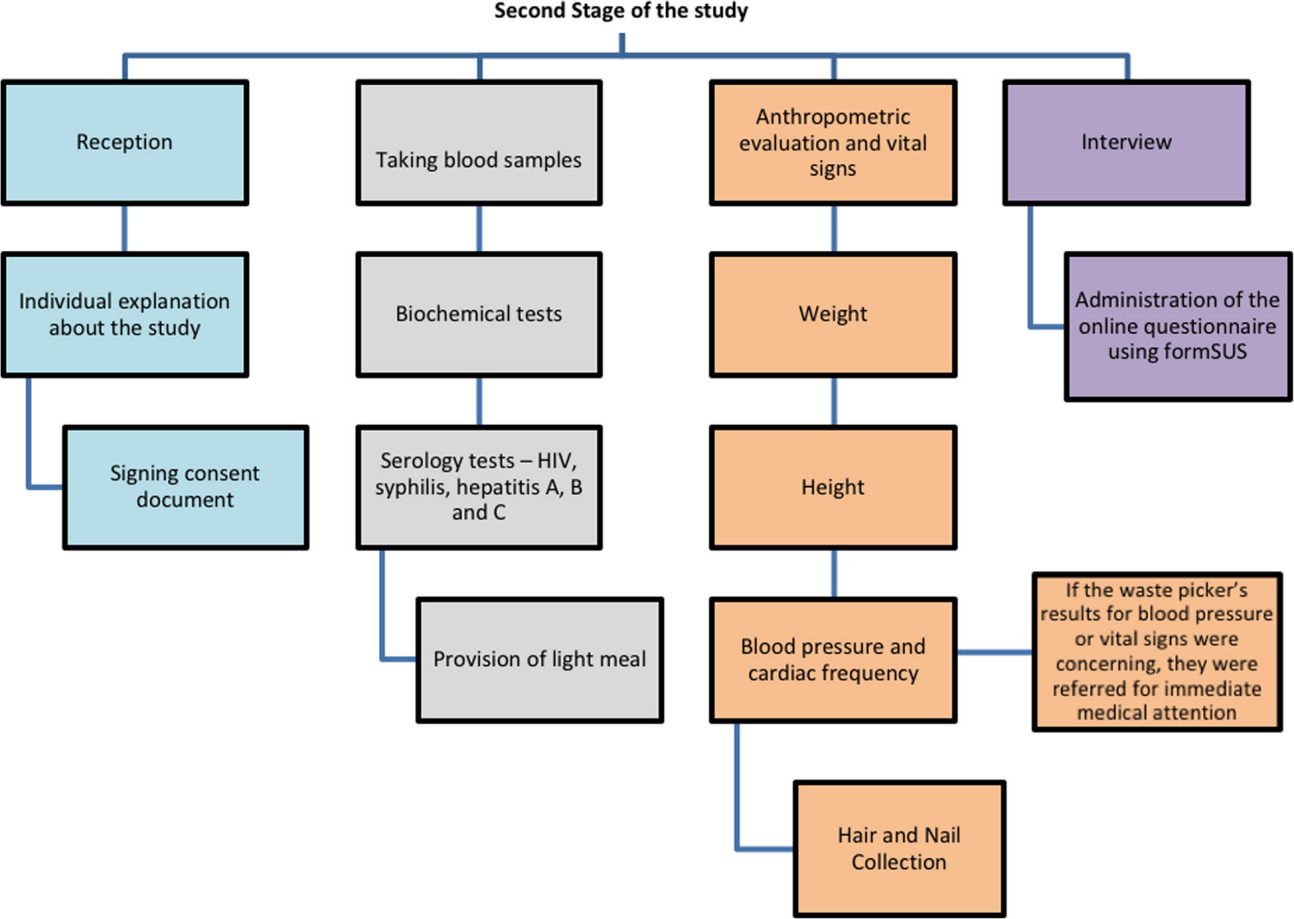
Second Stage of the study

All the reception procedures, as well as general advice on the health diagnosis, signing the terms of free and informed consent, blood, nail and hair collection, measuring weight and height to calculate Body Mass Index (BMI), and checking cardiac frequency and blood pressure were undertaken in a room in a primary healthcare center close to their home. The interview was undertaken individually in a designated area in the health center itself, to ensure the interviewees’ privacy. Figure 2 represents the description of the study related to the second stage.

### Reception

The waste pickers were received by the project team and receive an explanation about the study. Their doubts were clarified and they sign the terms of free and informed consent after reading them and receiving an explanation of the same.

### Blood collection

After that, blood samples were taken and the participants received a light meal (as they had been fasting for at least 8 h). The biochemical tests requested were: blood sugar, complete blood count, urea, creatinine, uric acid, serum glutamic oxaloacetic transaminase (SGOT), serum glutamic-pyruvic transaminase (SGPT) Gamma GT and lipid profile, Total Cholesterol, high density lipoprotein (HDL), very-low-density lipoprotein (VLDL), low-density lipoprotein (LDL) and Triglycerides. Method: enzymatic colorimetric.

Because of the occupational hazards related to working in open garbage dumps in Brazil – probably broadly identical to those found in similar sites elsewhere in the world [18] we propose the inclusion of biological markers for parenterally-transmitted diseases spread through cuts.

The serological screening for the infectious diseases selected is for: viral hepatitis type B (HBV), HCV, HIV and *T. pallidum* and the screening included the HBsAg, and HIV-1 p24 antigen; and for antibodies against HBV (anti-HBc and Anti-HBs), HCV (anti-HCV, for HCV core antigen, NS3Ag, NS4Ag, and NS5-Ag) and HIV (anti-HIV-1/2, HIV-1 group O).

The provision of a light meal after the blood test is fundamental, as the workers had not eaten for several hours beforehand, and most of them intended to head directly to work after the blood samples were taken and the interview conducted. In this study, the meals were donated by SESI (*Serviço Social da Industria* – the Industry Social Service) of the Federal District.

### Hair and nail sampling for identifying metal toxicity

When the waste pickers returned to give blood samples, after having a light meal, they were invited to give hair and nail samples to be tested for metal exposure. The hair collection followed the Society of Hair Testing’s “Recommendations for Hair Testing in Forensic Cases” as well as the recommendations provided by the European Guidelines for Workplace Drug and Alcohol Testing in Hair [31]. Briefly, a sample should be taken from the posterior vertex region of the head, as close as possible to the scalp, as growth rate varies least in this region. If not, the source of the sampling should be described. In general, head hair is estimated to grow at approximately 1.0 cm per month. The presence of heavy metals in the hair is a relevant biomarker for exposure to these substances, and the concentrations are predictive of toxic effects [32]. Heavy metals may be bio accumulative and potentially toxic [33]. The main heavy metals that present toxicity is aluminum, lead, copper, chromium, tin, nickel, mercury, vanadium and zinc. Residues containing cadmium, manganese, chromium and nickel can cause dermatitis, skin ulcerations, cancers, affective disorders, neuromuscular irritation and headache, and are suspected to affect the immune system [34].Nail analysis for metal detection has been widely used in forensic medicine but also in general and environmental medicine for diagnosis and prevention of disease. Interest in this kind of human sample as a biomarker for metal exposure is based on the fact that many elements bind to keratin, the proteins that contain SH bonds present in the nails [35].

Description of the steps: Collection and storage of nail samples- simple, well-sanitized nail clippers were used and about 1 g of each person’s nail was collected for analysis and stored in Falcon tubes at room temperature; Sample washing- samples were washed to remove any dirt. In the case of hair: 0.5 g successively: 1 time with acetone, 3 times with water and 1 time with acetone; between each wash, the hair was left covered with the solvent at room temperature. The hair was left to dry over night wrapped in chromatographic grade filter paper [36]. Nail samples were soaked in 1% Triton and acetone and dried in a laminar flow chamber; Digestion of samples- after drying and weighing, the samples were dissolved with tetra methylammonium hydroxide (TMAH) in a microwave oven [36, 37]; Sample analysis- the samples will be analyzed by inductively coupled plasma mass spectrometry (ICP-MS) according to the method proposed and validated by BATISTA and collaborators (2008), being rhodium (Rh) added as an internal standard [37].

### Anthropometric evaluation and vital signs

After taking the blood sample, the waste pickers received a light meal (as they had been fasting for at least 8 h) and were sent for anthropometric evaluation, which consisted of measuring their weight and height to establish their body mass index (BMI). The measurements taken include checking participants’ body weight (in kilograms) on digital scales (Líder®) and their height (in meters) using a stadiometer (Líder®), the procedures being undertaken in accordance with techniques stipulated by the Brazilian Association for the Study of Obesity and Metabolic Syndrome [38]. The variable of Body Mass Index (BMI) in this study was classified according to the Brazilian Guidelines on Obesity, of the Brazilian Association for the Study of Obesity and Metabolic Syndrome [38]. To assess BMI, in which weight (kg) is divided by height squared (m2), the reference of the Brazilian Association for the Study of Obesity and Metabolic Syndrome was used [38].

Physician scales, made up of an electronic system with a six-digit LED display, with capacity up to 150 kg, were used. The measurement was taken on the left arm, with a standard cuff with a width of 12 cm, indicated for circumferences of around 30 cm [39]. The cuff was placed firmly 2 to 3 cm above the elbow, centralizing the middle of the rubber bladder above the brachial artery. With the stethoscope in the ears, with the bell on the outside, the brachial artery was palpated on the front part of the elbow and the stethoscope’s bell was held in position lightly. The cuff was inflated rapidly until it passed the estimated level of SAP (systolic arterial pressure) by 20 to 30 mmHg, obtained by palpation. In order to estimate the level of SAP, the radial pulse was palpated and the cuff was inflated until the pulse ceased to be felt under the fingers. This was the estimated level for the SAP: at this point, the cuff was inflated a further 20 to 30 mmHg. SAP is noted at the point determined by the first audible sound, this being a weak sound followed by regular beats which increase in intensity. DBP (Diastolic Blood Pressure) is considered as the point at which the sounds disappear, that is, when they cease to be audible. The deflation (emptying) of the sphygmomanometer was slow, at a velocity of around 2 to 4 mmHg per second [39]. The measurement of the blood pressure was undertaken using an aneroid sphygmomanometer (Missouri MikatosR – model 102-NYL) and Rappaport stethoscope (SupermedyR).

If the waste picker had blood pressure levels outside normal parameters, he or she was immediately attended by the physician or nurse from the team they were covered by. When this was not possible, he or she was attended by the physician and/or nurse on duty from the primary health care center and was later referred to the relevant team.

### Interview

The waste pickers were invited to be interviewed. These were undertaken individually by their searchers in reserved areas of the Health Center. Interviews lasted approximately 20 min. For data collection, a structured questionnaire was developed by the study’s team of researchers. The questionnaire used was organized using the FormSUS format [40]. The instrument was used with the participants in the form of an interview and the answers were saved on tablets.

Before the questionnaire was administered, it was validated in the first week prior to the undertaking of the study in a sample less than 10%, which was later discarded, this being an opportunity for all of the interviewers to familiarize themselves with the instrument before the fieldwork began, as well as to identify the understanding of the questions in the questionnaire by the participants. The questionnaire is divided into 7 blocks, as described in Table 1. The complete version is in Additional file 1: entitled: Water, the Environment and Health: impact on the living conditions of waste pickers.

**Table 1:** Questionnaire topics, variables and number of questions

Topics of the Questionnaire	Variables	Number of questions
Demographic status	age, sex, race/color, number of children	24
Socioeconomic status	income, educational level, marital status, housing conditions	14
Lifestyle	sexual behavior, use of illicit substances, leisure, physical activities	19
Nutrition	type of food consumed	9
Work conditions	Time in this job, place of work, type of material handled, exposure to risks, use of personal protective equipment, occupation al accidents	52
Health conditions mentioned	diabetes, hypertension, cancer, kidney diseases, dermatological diseases, respiratory diseases, allergies, waterborne diseases and mental disorders	91
Access to the health services	medications, tests, primary and secondary care	21

#### Stage 3—the return of the waste pickers to receive the results of the tests and report occupational accidents (knowledge translation and exchange- KTE)

In this stage, the waste pickers who underwent the tests are being called in by their respective family health teams to receive the results, and, according to their needs, are being treated in primary care. If necessary, they are referred to a more complex level of care.

Also in this stage, if the waste picker has suffered a serious work-related accident or has been exposed to biological waste, more detailed information is collected about the accident so that it may be recorded on the Occupational Accidents Surveillance System of the National Information System of Notifiable Diseases and Injuries (SINAN- *Acidentes de trabalho*), Ministry of Health, Brazil.

After this initial diagnosis, the family health team to which the waste picker is linked according to his or her place of residence is to monitor his / her health conditions. Some tests not covered in the initial stages for the waste pickers can be requested by the family health care team in specific situations when there are symptoms, a clinical history or a positive or strong epidemiological factor, as long as the tests are available, and can be undertaken by the Federal District’s Health Laboratory Network.

### Statistical analyses

A descriptive and analytical analysis of the database supported by the Epi Info [41] and R [42] software will be conducted. The data will be summarized using absolute numbers, proportion, and measures of tendency and dispersion. We will compare the workers’ social and demographic characteristics and work conditions with their health outcomes and occupational diseases. After this step we will compare data on exposure collected by a standardized questionnaire using, as a measure of association, the prevalence odds ratio (and 95% confidence intervals) with the waste pickers without diseases (called: internal control group - 1). Furthermore, these two observational epidemiological studies will provide additional information that could potentially be related to the labor activities.

We will use the Chi-square test (or Fisher’s exact test, if expected value is under 5) to compare the statistical differences for categorical variables, and we will use the Student’s t test (or the Wilcoxon signed-rank test) for continuous variables of the database. We will conduct a multivariate analysis using Poisson regression to minimize confounders. For this analysis, all variables with *p* value < 0.20 in the bivariate analysis will be included in the matrix correlation (R2 Pearson) to identify collinearity. In addition, a stepwise selection in a backward approach will be used to measure independent β by each variable selected for inclusion in the model.

## Results

During the first and second stages of this protocol, 1083 waste pickers were included in the study and referred to the family health teams by which they were covered. In all, 1025 waste pickers undertook tests and interviews, following the proposed protocol, representing 94.6% of the total who worked in the open dump. Those who undertook the interview alone formed 2.4% of the total, and those who undertook the blood tests alone formed 2.9% of the total.

This investigation identified about the demographic and socioeconomic status that 67.0% of the participants were female, most of the workers were between 36 and 45 years old (45.7%) followed by over 45 years old (36.4%) and under 35 years old (18.0%). Besides age and gender, other demographics were reviewed. In terms of ethnicity, most participants were brown or mixed race (62.8%); black (24.9%); white (11.7%); yellow (0.2%) and indigenous (0.4%). According to marital status, most of them were single (58.1%) or divorced (7.5%). A total of 96% of participants had children. An overall of 66.0% of participants appear to be single parents. In men group, majority of participants with 46.0% had between 1 to 2 children while majority of women had had between 3 to 5 (48.0%). In total, 27.3% of the participants did not attend to any school, 47.7% were educated only up to primary level and among them, 44.0% were not able to read and write.

Of this population, 16.9% were living in marginalized (slum areas), 10.0% did not have public water supply and 27.0% lived in households that were not connected to sewer system. Among households with access to public water supply, 64.0% consumed water with no additional treatment, while only 34.0% used filtered before water consumption. Regarding lifestyle, 28.0% of the total were smokers; 22.7% consumed alcohol more than three times a week; 14.8% reported the use of psychotropic’s and 13.2% had history of psychological or psychiatric treatment. There were higher rates of smokers and alcoholic in men (31.6%; 32.7%) than women (25.0%; 17.8%), respectively.

For the workplace, 85.9% have worked exclusively in the massif of the dumpsite, whereas 14.1% were used to work there and on the streets or in sheds too. When it comes to activity type, 82.1% of participants worked with bags, mats or presses, whereas 17.9% worked manually or with trolleys. In addition, 61.9% of participants worked only in the day shift (from 8 am to 6 pm), whereas 19.4% worked at night and 18.7% mixed (day and night) shifts; 48.6% of waste pickers worked between 5 to 8 h a day, whereas 44.5% worked more than 8 h a day and only 6.9% up to 5 h a day. The waste picking activity was the only source of income for 77.6% of participants. Many of these people have been working as recyclable collectors for most of their working life with an average of 15 years.

In this study, the majority of waste pickers (68.7%) reported accidents and most of them (89.7%) were caused by glass, cans and needles during their work activities even though they used gloves, boots and long-sleeved shirts as Personal Protection Equipment. According their responses, the most referred diseases were: osteomuscular disorders (78.7%); arboviruses (28.6%); episodic diarrhea (24.9%); hypertension (24.2%); bronchitis (14.3%); intestinal worms (12.6%) and diabetes (10.1%). Regarding to the anthropometric measure, 32.6% were overweight and 21.1% were obese. Only 29.9% did some physical activity out of work. About nutrition, 86.6% reported three or more meals per day, but at least one of them were done inside the open dump.

According to the blood tests, the values outside the reference limits were: Uric acid (23.9%); creatinine (54.1%); GGT range (16.0%); SGOT - Serum Glutamic Oxalacetic Transaminase (5.3%); SGPT- Serum Glutamic Pyruvate Transaminase; glucose (35.5%). According to serology’s there were 28 positive cases of syphilis, 6 cases of HIV/AIDS infections and 33 cases of hepatitis B as described in Table 2.

**Table 2:** Blood Test Results

Exam Group Description	Reference value	Values within the reference limits		Values outside the reference limits		Total
		n	%	n	%	
Uric acid	3.5–7.2	586	76.10	184	23.89	770
Creatinine	0.80–1.40	356	45.93	419	54.06	775
GT Range	10.0–49.0	634	83.75	123	16.24	757
TGO (Oxalacetic Glutamic Transaminase)	0–38	733	94.70	41	5.29	774
TGP (Transaminase Glutamic Pyruvate)	0–41	701	90.56	73	9.43	774
Glucose	70–99	499	64,47	275	35.52	774
VDRL (Inclusive Quantitative)	Non reactive	654	95.89	28	4.10	682
Antic. Anti-hiv1 + Hiv2 (elisa)	Non reactive	764	99.22	6	0.77	770
HIV1 / 2 Rapid Immunoblot	Non reactive	4	57.14	3	0.42	7
Hepatitis B	Non reactive	737	95.70	33	4.30	770

In relation to stage 3 of the study, at the time of writing, a total of 879 waste pickers have been attended by their family health teams (85.8%). This attendance is related to the results of the laboratory tests undertaken and seeks to link the waste picker with the relevant health team. The other waste pickers are being contacted for follow-up treatment.

## Discussion

It is known that vulnerable populations who manage waste, such as the waste pickers, are exposed to many occupational risks including chemical hazards, infection, musculoskeletal damage, mechanical trauma, emotional vulnerabilities, and environmental contamination [43]. This is the first study of this type in Brazil, and possibly in the world, in which all waste pickers in an open dump were invited to participate; a sampling process was undertaken, involving detailed interviews with 222 questions on these workers’ health, working and living conditions, as well as biochemical tests, serology, anthropometric evaluation, blood pressure and vital signs.

In this study, we found a high prevalence of osteomuscular disorders (78.7%); arboviruses (28.6%); episodic diarrhea (24.9%); hypertension (24.2%); bronchitis (14.3%); intestinal worms (12.6%) and diabetes (10.1%). In a systematic review, formal and informal solid waste workers in Brazil reported some sort of pain or discomfort in the limbs and back. During their work they are constantly exposed to squatting, vibration, awkward postures, and repetitive movements [43]. Other studies reported waterborne diseases including arboviruses, diarrhea, worms or/ and chronic illness as diabetes, hypertension and respiratory diseases [7–11]. Our study coincides with the national findings of prevalence of hypertension, diabetes, changes in cholesterol and obesity in the Brazilian population [49]. However, the scavengers presented higher rates of these diseases, which can be explained and aggravated by the work environment to which they are subjected besides social vulnerability. Highlight for the musculoskeletal, respiratory and renal diseases identified in the study and could have a direct relation with their work activity [44].

The precarious work conditions, along with socio-economic and psychological stress, and exposure to different forms of violence, are also factors that impact workers’ health and overall well-being. Most of these workers are under informal employment without access to social protection where on average, earnings are low and risks are high [43]. The population of this study was not different: most of them were women, with many children, single or divorced, low level of education, working most time of their lives as waste pickers, with low income and living in poor areas with lack of sanitation and infrastructure. Some studies in Brazil and in other countries found some similar rates of age and level education between the workers showing social vulnerability and the influence of low level education to choose this activity [11, 12, 44, 46, 47]. The workers in this study had few job options other than waste picking due to their high illiteracy and low education level.

According to race, the data of this study presents the profile of the poorest Brazilian population, with racial miscegenation [45]. The results were different in other study fulfilled in the South of Brazil where there are more white people living there [46]. According to another study undertaken with waste pickers at the Estrutural Garbage Dump, the majority of the workers were women [47] and this result has been found in other locations in Brazil [48]. Women’s significant presence in this profession may be explained by their need simply to survive. Many are raising their children alone and are too poorly-educated to obtain better work. As a result, working as a waste picker with solid waste appears as an alternative for women faced with unemployment or exclusion from the formal job market. It makes it possible to carry out the role of health surveillance, undertaking an active search for individuals presenting vulnerabilities and at risk of developing diseases – and to listen to their complaints, and show society the importance of these professionals.

The exhausting conditions of this work and the low level of income are also other concerns regarding this job. In this study, most of them worked as waste pickers for 15 years, between 5 or more hours a day, 6 days a week and income of the workers appeared to be below or up to minimum wage, which comes to about US$ 250. Other study undertaken in Brazil with waste pickers also found poor living conditions, low levels of sanitary supply, education and income with waste pickers who worked on the streets. However, they worked an average on this job for 3 years, and 6 h/day [46].

Environmental aspects related to housing and work conditions also contributed to a high prevalence of diseases in these workers. In our study, almost 30% of the workers reported waterborne diseases. These findings show that the sanitary conditions of the site and the area around, the lack of access to treated water in some neighbourhoods of the Estrutural and the inadequate storage of available resources act as facilitating factors for the rapid multiplication of waterborne diseases and vectors such as *Aedes aegypti*. Regarding housing conditions and socioeconomic status, a systematic review with waste pickers in Brazil indicated that most workers lived in rental houses, while others resided in non-permanent housing or communities that lacked basic infrastructure and quality sanitation; some workers had admitted to living in the streets or on the waste collection sites where they worked, near the open dumps; lived illegally or squatted and some recyclable collectors lived in their own residences, but these were often provided by the city administration and social programs [44].

Regarding lifestyle, we found higher rates of smokers and alcohol consumption in comparison to Brazilian investigation while smokers in the population above 18 years of age in Brazil in 2017 were 10.1% (men 13.2% and women 7.5%) and heavy drinkers were 19.1% (men 27.1% and women 12.2%) [49]. The social exclusion and prejudice with this profession associated to financial difficulty act as stimulators of these alternative habits for sense of well-being and pleasure impacting mental health [43, 46]. The consumption of illicit drugs and the history of psychiatric treatment was also high, pointing the mental disorder as one of the health problems in these workers [44].

According to the blood tests in this study, the main values outside the reference limits were: Uric acid; creatinine; GT range and glucose (Table 2). Many of the waste pickers have been working as recyclable collectors for most of their life. This amount of time suggests chronic exposure levels from possible contaminants and hazards but to affirm this association it would be necessary to follow up with these workers during the time to identify the period of manifestation of the disease.

On other hand, this study showed a high prevalence of accidents during work in accordance with many studies which also reported accidents and most of them related to sharp objects including needles, syringes, among other wastes [10, 11, 43, 44, 46]. Many of these injuries are caused by inadequate storage of trash [46]. Since they are often exposed to materials contaminated with biological waste from the health services or home care, they are exposed to diseases related to (i) sharps injuries, including injuries involving sharps contaminated with etiological agents such as HIV and HCV [50] (although it is illegal for hospital waste to be discarded without sterilization in open-air dumps, due to poor control, this does happen); and(ii) residues containing mercury, lead, cadmium, and other causes of toxicity. In addition, there are other ways to be contaminated by these diseases and studies have demonstrated that more socially vulnerable populations, such as the one in question (waste pickers) may be at greater risk of contamination [43, 44, 51].

A study undertaken in Brasilia, Distrito Federal analyzed the drug disposal form by the population. Among the 393 interviewees, 73.6% had already discarded drugs in the commom waste. It presents the need to incorporate the theme into specific policies and media [14]. Other measures required include the improving of selective collection through strengthening environmental education and the installation of open skips and recycling bins, where residents are encouraged to place garbage around the city including appropriate facilities to discard the medical waste correctly. It is hoped that the waste arriving in the recycling sorting facilities correctly separated, will expose the waste pickers to fewer risks and thus protecting their health.

The biochemical tests undertaken in this study for blood sugar, complete blood count, creatinine, uric acid, SGOT, SGPT, GGT, lipid profile, total cholesterol and cholesterol fractions and triglycerides – were very important to make it possible to map both results which are outside normal parameters and risks of developing chronic diseases in the future which would make it impossible for them to work and harm their quality of life [52].

Measuring the anthropometric evaluation, blood pressure and vital signs made it possible for those waste pickers presenting results outside normal parameters to be referred immediately for an appointment with a physician, or to be treated and monitored by their family health team.

Besides the general biochemical tests, serology tests were also proposed in this protocol for viral hepatitis (types B – HBV and HCV), HIV and *T. pallidum*. Recyclable collectors were also exposed to communicable diseases, including sexually transmitted infections. According to serology’s there were 28 positive cases of syphilis, 6 cases of HIV/AIDS infections and 33 cases of hepatitis B. The study was extremely important to identify the cases allowing the worker to know about their health condition and receive timely treatment within the basic care for their protection and prevention of their communicants. Regarding *T. pallidum*, an epidemic of syphilis has been observed in Brazil in recent years, aggravated by the worldwide shortage of penicillin; health surveillance data have shown an increase in infection in adults, as well as the vertical transmission of this disease [53]. As this disease too is related to poverty, and is a neglected disease, the researchers considered its evaluation in this population to be important [54]. The results allow health professionals to offer the patients immediate treatment and prevent further contagions, thus protecting their partners and families. Many waste pickers who worked in the Estrutural Garbage Dump stated that they had never had a laboratory test in their lives.

In addition to the environmental impact caused by the presence of a dump, the direct handling of the waste - especially batteries and electronic waste- offers physical and chemical risks to the workers employed there. Among the chemical risks are metals that come from the solid waste in the dump and which can contaminate the population not only directly, through contact, but indirectly, through water, air, soil and food. The heavy metals can be bio-accumulative, potentially toxic, and can provoke dermatitis, ulcerations on the skin, cancers, affective disorders, neuromuscular irritation, and cephalgia [34, 43]. The community of waste pickers is largely unaware of the severity of the risks posed by the chemicals to which they are exposed, which means that - through its analysis of nail and hair samples, identifying and quantifying the metals and other products to which the individuals are exposed – this investigation, through health education actions, can raise their awareness of the risks they face and thereby reduce health impacts. In this case, they can be exposed to toxic metals by managing e-waste and consuming contaminated water. A systematic described high levels of lead in the blood of recyclers working in landfills, leading researchers to associate their work with increased bioaccumulation [43]. As these workers are women in majority, a special concern is about their children during pregnancy and breast feeding. Lead and dioxin related-compounds were discovered in higher concentration within the breast milk of women neighboring landfills of recycler communities [43]. It has been established that toxicity and absorption of these metals is elevated in children under the age of 6 years compared to adults due to their not having fully developed nervous system and other organs [55]. In addition to lead, mercury and cadmium are of serious concern. They can develop neurodevelopment disorders, and other grave diseases. For now, 645 waste pickers agreed to collect and donate the hair and nail samples, which are being analyzed until the moment of writing this article.

While carrying out the study, the group was concerned to treat the waste pickers with great respect and to show admiration for their work. We were aware that they face great prejudice from society and that they are often treated as inferior. This situation needs to change, starting with the health teams who attend them. Meetings were held monthly with the Family Health Strategy teams working in the primary health care center in Estrutural, to raise these professionals’ awareness about the importance of the study and of the partnership between the University, service and community.

The meetings with the ten Family Health Strategy teams that attend the waste pickers were characterized by dialogue about the need for professionals geared toward health promotion and work with communities, attuned to the population’s needs and focused not simply on treating individuals, with a clinical perspective restricted to techniques, but, rather, prepared to meet the needs of the people, their families and the community – and prepared to change the paradigm of care.

Creating a bond between the study team and the waste pickers made it possible to build a relationship of trust and led to greater adherence in the study. Every day, before beginning the epidemiological diagnosis, the researchers explained the study’s aims to the participants and listened to their expectations and questions. When the waste picker presented some health complaint not covered by the study, the team made an effort to refer the participant or provide guidance so that the problem could be resolved. The integration of teaching, service and community made it possible for the collective work and teamwork to be undertaken successfully, integrating students and professors from undergraduate courses in the health area with the workers who make up the teams from the health services, including the managers, with a view to improving the quality of the individual and collective healthcare and the quality of the professional training, as well as contributing to the development/satisfaction of the workers from the services and of the waste pickers who are users of the Unified Health System.

One result of this project was the extension of the coverage of the Family Health Strategy in *Cidade Estrutural*, with physicians being sent to work in the area of Santa Luzia, which was previously not covered by the Family Health Strategy. This area is fairly close to the garbage dump. This move will broaden the care provided to this population.

The waste pickers were highly interested in participating in this health diagnosis, and many participated. This reflects these workers’ significant vulnerability in relation to their health needs. Another factor which facilitates the participation is that most of the waste pickers are women, who take more interest in their health than men do.

In addition to the diagnosis of the waste pickers’ health undertaken by the University of Brasilia (UnB) School of Health Sciences and State Secretariat of Health of the Federal District, Brasília, Brazil, other actions are being carried out by the government of Brasília to improve these workers’ living conditions. It is hoped that the waste pickers’ income and working conditions will improve, to compensate them for the reduction in the demand for waste during this transition phase, as they move from the old dump, as it is closed down, to new selective collection centers. Those registered in the cooperatives, working in the separation areas, will have the right to receive monthly financial help for a limited period. In addition to the values received by the group for selling the separated materials, the government will make further payments based on the number of tons sold, to meet the costs of their INSS (Social Security) and their protection equipment, as well as to maintain the area in a hygienic condition.

The study’s limitations relate in particular to the fact that it is not possible to define causal links between the health conditions observed and the waste pickers’ living and work conditions. This limitation could be minimized only by studies of the cohort or case-control type. The findings suggest the need for further qualitative research with informal recyclers to understand better the occupational risks, job perception and difficulties to enforce the importance of this job by the solid waste policy makers and society.

## Conclusions

In the light of the context of vulnerability that the waste pickers face, the publication of this protocol, and of this study’s results is of great importance, as it could indicate a means for investigating these individuals’ health and living and working conditions – and could be adopted in other garbage dumps in Brazil, as well as in other communities in situations of risk, and even in other countries facing similar contexts. This would allow more accurate comparisons and the monitoring of the groups investigated in various parts of the world. Investigating and publicizing the risk factors to which these workers are exposed could influence public policies, promoting the adopting of protective measures, and could raise society’s awareness so that correct habits for the discarding of waste might be acquired; above all, it could lead these professionals to be valued as true environmental agents.

## Additional file

Additional file 1: Questionnaire “Water, the Environment and Health: impact on the living conditions of waste pickers”. (PDF 639 kb)

## Abbreviations

BMI: Body Mass Index
CEMPRE: Compromisso Empresarial para a Reciclagem (Business Commitment to Recycling)
DBP: Diastolic Blood Pressure
FHS: Family Health Strategy
GGT: Gamma-Glutamyl Transferase
HBV: Viral hepatitis type B
HCV: Viral hepatitis type C
HDL: High density lipoprotein
HIV: Stands for human immunodeficiency virus
ICP-MS: Plasma mass spectrometry
LDL: Low-density lipoprotein
PNRS: Política Nacional de Resíduos Sólidos (National Solid Waste Policy)
SAP: Systolic arterial pressure
SDGs: Sustainable development goals
SESI: Serviço Social da Industria (the Industry Social Service)
SGOT: Serum glutamic oxaloacetic transaminase
SGPT: Serum glutamic-pyruvic transaminase
SINAN: National Information System of Notifiable Diseases and Injuries
SLU: Urban Cleaning Service
SSDO: South Sudanese Development Organization
SUS: Sistema Único de Saúde (Unified Health System)
TMAH: Tetra methylammonium hydroxide
UnB: University of Brasilia
VLDL: Very-low-density lipoprotein

## References

[1] Hoornweg D, Bhada-Tata P. What a waste: a global review of solid waste management. 1st edition. Washington, DC, USA: Urban Development & Local Government Unit; 2012. www.worldbank.org/urban.

[2] ABRELPE. Panorama 2017: Resíduos Sólidos Urbanos. Abrelpe. 2018. p. 74. http://abrelpe.org.br/pdfs/panorama/panorama_abrelpe_2017.pdf. Accessed 13 Feb 2018.

[3] Guerrero LA, Maas G, Hogland W (2013). Solid waste management challenges for cities in developing countries. Waste Manag.

[4] Gutberlet J, Baeder A, Pontuschka N, Felipone S, dos Santos T (2013). Participatory research revealing the work and Occupational Health hazards of cooperative recyclers in Brazil. Int J Environ Res Public Health.

[5] Cointreau S. Occupational and Environmental Health Issues of Solid Waste Management Special Emphasis on Middle- and Lower-Income Countries. 1st edition. Washington, DC, USA: The International Bank for Reconstruction and Development/The World Bank; 2006. http://www.worldbank.org/urban/.

[6] Ziraba AK, Haregu TN, Mberu B (2016). A review and framework for understanding the potential impact of poor solid waste management on health in developing countries. Arch Public Heal.

[7] Chokhandre P, Singh S, Kashyap GC (2017). Prevalence, predictors and economic burden of morbidities among waste-pickers of Mumbai, India: a cross-sectional study. J Occup Med Toxicol.

[8] Jayakrishnan T, Jeeja M, Bhaskar R (2013). Occupational health problems of municipal solid waste management workers in India. Int J Environ Health Eng.

[9] Mol MP, Pereira AF, Greco DB, Cairncross S, Heller L (2017). Assessment of work-related accidents associated with waste handling in Belo Horizonte (Brazil). Waste Manag Res.

[10] Cowing MJ. Health and Safety Guidelines for Waste Pickers in South Sudan. 1st edition. South Sudan: United Nations Environment Programme: South Sudan; 2013. http://unep.org/SouthSudan/.

[11] Thakur P, Ganguly R, Dhulia A (2018). Occupational Health Hazard exposure among municipal solid waste workers in Himachal Pradesh, India. Waste Manag.

[12] Fazzo L, Minichilli F, Santoro M, Ceccarini A, Della Seta M, Bianchi F (2017). Hazardous waste and health impact: a systematic review of the scientific literature. Environ Health.

[13] Mitis F, Martuzzi M. Population health and waste management: scientific data and policy options. 1st edition. Copenhagen, Denmark: WHO Regional Office for Europe; 2007. http://www.euro.who.int/__data/assets/pdf_file/0012/91101/E91021.pdf. Accessed 13 Feb 2018.

[14] RAMOS HAYSSA MORAES PINTEL, CRUVINEL VANESSA RESENDE NOGUEIRA, MEINERS MICHELINE MARIE MILWARD DE AZEVEDO, QUEIROZ CAMILA ARAÚJO, GALATO DAYANI (2017). MEDICATION DISPOSAL: A REFLECTION ABOUT POSSIBLE SANITARY AND ENVIRONMENTAL RISKS. Ambiente & Sociedade.

[15] WIEGO. Waste Pickers Around the World Database | WIEGO. http://www.wiego.org/resources/waste-pickers-around-world-database. Accessed 16 Feb 2018.

[16] Porta D, Milani S, Lazzarino AI, Perucci CA, Forastiere F (2009). Systematic review of epidemiological studies on health effects associated with management of solid waste. Environ Health.

[17] Galon T, PHM M. Condições de trabalho e saúde de catadores de materiais recicláveis na América Latina: Uma revisão. In: BCJ P, Goes FL, editors. Catadores de Materiais Recicláveis – um encontro nacional. 1st edition. Rio de Janeiro: Ipea; 2016. p. 169–99. http://www.ipea.gov.br/portal/images/stories/PDFs/livros/livros/160331_livro_catadores.pdf.

[18] Ferreira RGPS, da Silva TC, Ramalho WM, Araújo WN, Nogueira CVR. Condições de saúde e estilo de vida dos catadores de resíduos sólidos de uma cooperativa da Ceilândia, no Distrito Federal: Um olhar acerca dos determinantes sociais e ambientais de saúde. In: Pereira BCJ, Goes FL, editors. Catadores de Materiais Recicláveis – um encontro nacional. 1st edition. Rio de Janeiro. Brazil: Ipea; 2016. p. 151–68. http://www.ipea.gov.br/portal/images/stories/PDFs/livros/livros/160331_livro_catadores.pdf.

[19] Medina M. Solid wastes, poverty and the environment in developing country cities: challenges and opportunities. UNU-WIDER World Institute for Development Economics Research: Helsinki, Finland; 2010. p. 1-17. https://www.wider.unu.edu/sites/default/files/wp2010-23.pdf. Accessed 13 Feb 2018.

[20] United Nations. Water and Sanitation - United Nations Sustainable Development. http://www.un.org/sustainabledevelopment/water-and-sanitation/. Accessed 21 Feb 2018.

[21] Dominguez AGD, Cruvinel VRN. A Política Nacional de Resíduos Sólidos no Brasil e o Papel do Catador: Avanços e Desafios. In: Xavier L de O, Avila CFD, editors. Cidadania, Direitos Humanos e Políticas Públicas no Brasil. Estudos e Pesquisas Pós-graduadas. 1st edition. Curitiba, Brasil: Editora CRV; 2016. p. 375–90.

[22] Associação Brasileira de Empresas de Limpeza Pública e Resíduos Especiais. Panorama dos resíduos sólidos no Brasil 2016. 1st edition. São Paulo: Abrelpe; 2016. http://www.mpdft.mp.br/portal/pdf/comunicacao/junho_2018/panoramaanexos2016.pdf. Accessed 13 Feb 2018.

[23] Brasil (2017). Política Nacional de Resíduos Sólidos. 3rd edition.

[24] Silva SP, Goes FL, Alvarez AR. Situação social das catadoras e dos catadores de material reciclável e reutilizável. Brasil. 1st edition. Brasília: Instituto de Pesquisa Econômica Aplicada; 2013. http://www.ipea.gov.br/portal/images/stories/PDFs/situacao_social/131219_relatorio_situacaosocial_mat_reciclavel_brasil.pdf. Accessed 13 Feb 2018.

[25] Paim J, Travassos C, Almeida C, Bahia L, Macinko J (2011). The Brazilian health system: history, advances, and challenges. Lancet (London, England).

[26] Marten R, McIntyre D, Travassos C, Shishkin S, Longde W, Reddy S (2014). An assessment of progress towards universal health coverage in Brazil, Russia, India, China, and South Africa (BRICS). Lancet..

[27] Macinko J, Harris MJ (2015). Brazil’s family Health strategy — delivering community-based primary care in a universal Health system. N Engl J Med.

[28] da Silva EN, Powell-Jackson T (2017). Does expanding primary healthcare improve hospital efficiency? Evidence from a panel analysis of avoidable hospitalisations in 5506 municipalities in Brazil, 2000–2014. BMJ Glob Heal.

[29] Serviço de Limpeza Urbana. Construindo un novo modelo de gestão dos resíduos sólidos do Distrito Federal, Relatório de atividades SLU, 2016. 1st edition. Brasília, DF, Brasil: Governo do Distrito Federal; 2017. http://www.slu.df.gov.br/wp-content/uploads/2018/11/relatorio_slus_2016.pdf. Accessed 16 Feb 2018.

[30] Codeplan. Companhia de Planejamento do Distrito Federal. http://www.codeplan.df.gov.br/. Accessed 21 Feb 2018.

[31] Salomone A, Tsanaclis L, Agius R, Kintz P, Baumgartner MR (2016). European guidelines for workplace drug and alcohol testing in hair. Drug Test Anal.

[32] Lakshmi Priya MD, Geetha A (2011). Level of trace elements (copper, zinc, magnesium and selenium) and toxic elements (lead and mercury) in the hair and nail of children with autism. Biol Trace Elem Res.

[33] Reddy DHK, Seshaiah K, Reddy AVR, Rao MM, Wang MC (2010). Biosorption of Pb2+ from aqueous solutions by Moringa oleifera bark: equilibrium and kinetic studies. J Hazard Mater.

[34] Jaishankar M, Tseten T, Anbalagan N, Mathew BB, Beeregowda KN (2014). Toxicity, mechanism and health effects of some heavy metals. Interdiscip Toxicol.

[35] Bakri S F Z, Hariri A, Ma’arop N F, Hussin N S A W (2017). Toenail as Non-invasive Biomarker in Metal Toxicity Measurement of Welding Fumes Exposure - A Review. IOP Conference Series: Materials Science and Engineering.

[36] Carneiro MF, Moresco MB, Chagas GR, de Oliveira Souza VC, Rhoden CR, Barbosa F (2011). Assessment of trace elements in scalp hair of a young urban population in Brazil. Biol Trace Elem Res.

[37] Batista BL, Rodrigues JL, Nunes JA, Tormen L, Curtius AJ, Barbosa F (2008). Simultaneous determination of Cd, Cu, Mn, Ni, Pb and Zn in nail samples by inductively coupled plasma mass spectrometry (ICP-MS) after tetramethylammonium hydroxide solubilization at room temperature: comparison with ETAAS. Talanta..

[38] Associação Brasileira para Estudo da Obesidade e da Síndrome Metabólica. ABESO - Associação Brasileira para Estudo da Obesidade e da Síndrome Metabólica. http://www.abeso.org.br/. Accessed 21 Feb 2018.

[39] Malachias M, Souza W, Plavnik F, Rodrigues C, Brandão A, Neves MFT, et al. 7^a^ Diretriz Brasileira de Hipertensão Arterial. Arq Bras Cardiol. 2016;107:1–83 http://publicacoes.cardiol.br/2014/diretrizes/2016/05_HIPERTENSAO_ARTERIAL.pdf. Accessed 21 Feb 2018.

[40] Brasil. Datasus. FormSus. http://formsus.datasus.gov.br/site/default.php. Accessed 21 Feb 2018.

[41] Software Epi Info. CDC/OMS version 7.2.2017 https://www.cdc.gov/epiinfo/support/por/pt_downloads.html (accessed Dec 13, 2017).

[42] R StudioTM [internet]. Version 3.4.3. Massachusetts: R Studio; c2016 https://www.rstudio.com/about/ (accessed 13 Dec 2017).

[43] Binion E, Gutberlet J (2012). The effects of handling solid waste on the wellbeing of informal and organized recyclers: a review of the literature. Int J Occup Environ Health.

[44] Zolnikov TR, da Silva RC, Tuesta AA, Marques CP, Cruvinel VRN (2018). Ineffective waste site closures in Brazil: a systematic review on continuing health conditions and occupational hazards of waste collectors. Waste Manag.

[45] IBGE-Instituto Brasileiro de Geografia e Estatística (2017). População chega a 205,5 milhões, com menos brancos e mais pardos e pretos. IBGE Notícias - Agência Brasil.

[46] Da Silva MC, Fassa AG, Siqueira CE, Kriebel D (2005). World at work: Brazilian ragpickers. Occup Environ Med.

[47] Cruvinel V, Araujo W, Martins C, Alvarenga J. Perfil dos Catadores de Resíduos Sólidos do Distrito Federal: Uma Análise Comparativa entre Associações de Ceilândia e Estrutural. Hegemonia – Rev Eletrônica Relações Int do Cent Univ Unieuro. 2017;19:67–87. http://www.unieuro.edu.br/sitenovo/revistas/revista_hegemonia_20/Vanessa%20Cruvinel%20e%20outros%20(5).pdf. Accessed 1 Mar 2018.

[48] de CJAB, Ramos NF, Alves CM, Forcellini FA, Graciolli OD (2013). Catadores de materiais recicláveis: análise das condições de trabalho e infraestrutura operacional no Sul, Sudeste e Nordeste do Brasil. Cien Saude Colet.

[49] Brasil, Saúde M da. Vigitel Brasil 2016. 2017. http://bvsms.saude.gov.br/bvs/publicacoes/vigitel_brasil_2016_saude_suplementar.pdf.

[50] Ream PS, Tipple AF, Barros DX, Souza AC, Pereira MS (2016). Biological risk among hospital housekeepers. Arch Environ Occup Health.

[51] Ribeiro BJ, Sousa BC, Carvalho CFA, Pimentel de AC, Lopes FG, Baima CJK, Malta LD, Lampe EM, Villar L. Cross-sectional study to determine the prevalence of hepatitis B and C virus infection in high risk groups in the northeast region of Brazil. Int J Environ Res Public Health. 2017, Jul 17;14(7). 10.3390/ijerph14070793 PMC5551231 28714924

[52] Vidigal LMHR, Viana L de G, Guatimosim P. Protocolos clínicos dos exames laboratoriais. 1st edition. Belo Horizonte, Brasil: Secretaria de Estado de Saúde de Minas Gerais; Universidade Federal de Minas Gerais; 2009.

[53] Brasil. Boletim Epidemiológico de Sífilis - 2017. http://www.aids.gov.br/pt-br/pub/2017/boletim-epidemiologico-de-sifilis-2017 (accessed 13 Dec 2017).

[54] Marks M, Solomon AW, Mabeya DC (2014). Endemic treponemal diseases. Trans R Soc Trop Med Hyg.

[55] Hussein WF, Njue W, Murungi J, Wanjau R (2008). Use of human nails as bio-indicators of heavy metals environmental exposure among school age children in Kenya. Sci Total Environ.

