# Supplementary material for: Health conditions and occupational risks in a novel group: waste pickers in the largest open garbage dump in Latin America
Source: BMC Public Health. 2019 May 16;19:581. doi: 10.1186/s12889-019-6879-x (PMC6521552; doi:10.1186/s12889-019-6879-x)
Supplement: Supplementary file 1 — Questionnaire “Water, the Environment and Health: impact on the living conditions of waste pickers”. (PDF 639 kb) [file 12889_2019_6879_MOESM1_ESM.pdf]

Water, the Environment and Health: impact on the living conditions of waste pickers

HEALTH QUESTIONNAIRE

*** Mandatory Field**
Important: **Please do not include any personal or private information in the fields marked "Visible to the Public".**

Click here if you have any questions about this form.

I. QUESTIONNAIRE IDENTIFICATION INFORMATION

1. Time interview started:

2. Time interview ended:

3. Interviewer's name:

4. Date:

5. Typed by:

6. Date typed:

II. PARTICIPANT'S PERSONAL DETAILS I am going to ask some questions about you

7. What is your full name (participant)? :

7.1 Federal District Public Health Card (SES/DF) No.:

7.2 Do you belong to a family healthcare team?

Yes
No

7.2.1 If yes, which one(s)?

01
02
03
04
05
06
07
08
09
10

8. What is your date of birth?

9. What state were you born in?

10. Gender:

0.Male
1.Female

11. How old are you? (full years):

12. Your skin color is... (Read the alternatives) :

1.White
2.Black

3. Pardo
4.Asian
5.Indigenous

13. What is your address (Street, Neighborhood, Municipality)?: :

14. Phone number (with area code):
Contact number for the head of the cooperative.

15. Landline:

16. Cell phone:

17. What is your marital status?

1.Single
2.Married
3.Divorced/Separated
4.Common-law marriage

18. How many children do you have?

19. How old are they?

20. Do any of them have a deformity / disability? (If the answer is no, go to question 23).

0.No
1.Yes

21. How many?

22. What kind of disability? :

23. If the participant is a man: has your wife/partner ever had a miscarriage? (If the answer is no, go to question 25).

0.No
1.Yes

24. If the answer is yes, how many? :

III – HOUSEHOLD INFORMATION Now I am going to ask some questions about your home:

25. What type of water supply do you have at home? (Read the alternatives):

1. Public water supply network
2.Well or spring on the property
3.Well or spring outside of the property
4.Tanker truck
5.Rainwater stored in a cistern
6.Rainwater stored some other way
7.Rivers, lakes and streams.

26. The drinking water in your home is:

1.Filtered
2.Boiled
3.Tap water
4.Treated in some other way at home
5.Well water

27. Is your home connected to the sewer system?

0.No
1.Yes

28. How is your water stored?

1. Open cistern
2. .Closed cistern
3. .Open water tank
4. .Closed water tank
5. .Other

29. Of other, please specify:

IV – LIFESTYLE HABITS Now I am going to ask a little about your habits and things you usually do

30. Do you smoke?
(If the answer is no, go to question 35).

0.No
1.Yes

31. Do you smoke every day? :

0.No
1.Yes

32. How many cigarettes do you smoke a day? : (One pack contains 20 cigarettes)

1. > 20
2. 10 to 20
3. 5 to 10
4. < 5

33. How long have you been a smoker?

34. How long has it been since you quit smoking?

35. Do you drink hard alcohol, beer or any other type of alcohol?
If the participant does not drink alcohol or does not know, go to question 37

0. No (confirm: "not even every now and then?")
1. Yes
2. Don't know
3. Occasionally

35.1 – How much do you drink?

36. How often do you drink alcohol?

1. Once a week
2. Twice a week
3. Three times a week
4. Every day

37. Do you use illegal drugs (psychotropics)?

0.NO
1.YES

38. Have you ever used illegal drugs (psychotropics)?
(If the answer is no, go to question 48).

0.NO
1.YES

39. Which one(s)? :

1. LSD
2. Marijuana
3. Crack
4. Cocaine
5. Other

40. If “other”, Please specify:

41. Have you ever been treated for drug addiction?

0. NO
1. YES

42. Which drug(s)? :

43. Are you currently using medication as a result of drug addiction?

0. NO
1. YES

44. Please specify:

45. Have you ever had psychological or psychiatric treatment (If the answer is no, go to question 47).

0. NO
1. YES

46. How long ago?

47. For how long?

48. Do you engage in physical activity for at least 30 minutes at a time in your free time? :
(for example: running, cycling, playing football)

0. NO
1. YES

49. How many days a week do you engage in physical activity?
No. of days

1. One to two
2. Three to four
3. Five or more

V – EATING HABITS Now I am going to ask about your eating habits

50. How many meals do you eat a day?

1. Breakfast
2. Mid-morning snack
3. Lunch
4. Afternoon snack
5. Dinner
6. Evening snack

51. Yesterday you ate (read the options):

51.1. Beans:

0. NO
1. YES

51.2. Rice:

0. NO
1. YES

51.3. Meat (beef, pork, chicken, fish and others) and eggs:

0. NO
1. YES

51.4. Fresh fruit (not fruit juice):

0. NO
1. YES

51.5. Vegetables (not potato, cassava or yams):

0. NO
1. YES

51.6. Hamburger and/or cured meats (ham, mortadella, salami, sausage):

0. NO
1. YES

51.7. Artificially Sweetened drinks (soda, juice box, powdered drink mix, boxed coconut water, , guaraná/redcurrant concentrate, sweetened fruit juice):

0. NO
1. YES

51.8. Instant noodles, savory snacks or salt crackers:

0. NO
1. YES

51.9. Junk food as cookie or candy (hard candy, lollipop, bubblegum, caramel candy, jello candy:

0. NO
1. YES

VI – WORK-RELATED INFORMATION Now I am going to ask about your work

52. Are you or have you ever been a waste picker?
(If the answer is no, go to question 101).

0. NO
1. YES

53. At what age did you start working as a waste picker?

54. How long have you worked as a waste picker?

55. If you are no longer a waste picker, how long ago did you stop?

56. Are you a member of a waste pickers` cooperative?

0. NO
1. YES

57. Which one?

58. Where do you work?

1. Open dumps
2. Sorting plants
3. On the street

59. How do you work? :

1.Bags
2. Conveyor belt
3. Compactor
4. Other

60. If other, please specify:

61. How many hours a week do you work? :

62. What time of day?

1. Daytime
2. Nighttime
3. Both

63. In general, how many hours a day do you work? :

1. Less than 5 hours
2. 5 to 8 hours
3. More than 8 hours

64. In the last week has anyone from your family helped or accompanied you waste picking?

0. NO
1. YES

65. Who?

1. Wife
2. Child
3. Husband
4. Father/Mother
5. Grandchild(ren)
6. Brother(s) / Sister(s)
7. Other

66. If other, please specify:

67. When working, are you exposed to sharp objects such as tin cans, nails and glass?

0. NO
1. YES

68. Are you at risk of being run over when working?

0. NO
1. YES

69. When working, do you use equipment that is worn or broken (e.g.: rusty knife, torn gloves, the same clothes several days in a row)?

0. NO
1. YES

70. What type of personal protective equipment (PPE) do you wear at work? :

1. Hat / cap
2. Raincoat
3. Gloves
4. Boots
5. Mask
6. Long-sleeved shirt

71. Does your work put you at risk of accidents?

0. NO
1. YES

72. Do you think your work is dangerous?

0. NO
1. YES

73. Do you consider your work exhausting?

0. NO
1. YES

74. Have you ever been injured while working?

0. NO
1. YES

75. If yes, how many times? :

76. What parts of your body were injured?

1. Head
2. Arms (except hands)

3 Hands
4. Legs (except feet)
5. Feet
6. Chest
7. Several body parts
8. Other

77. If other, please specify? :

78. What type of injury did you sustain?

1. Cut / puncture
2. bump / contusion
3. wound
4. fracture
5. lost limb / amputation
6. other

79. If other, please specify:

80. Did you seek treatment?

0. NO
1. YES

81. If the answer is yes, where did you go?

1. Healthcare clinic
2. Outpatient clinic
3. Emergency room
4. Hospital
5. Other

82. If other, please specify:

83. Was an Occupational Accident Report filled out?

0. NO
1. YES

84. Were you unable to work?

0. NO
1. YES

85. If the answer is yes, for how many days?

86. Do you pay social security tax (INSS)?

0. NO
1. YES

87. If yes, for how long? (in years):

88. Did you receive support from the cooperative where you worke(d) after your accident?

0. NO
1. YES

89. Did your injury make household chores or other everyday tasks difficult? :

0. NO
1. YES

90. Have you been on medical leave in the last 12 months (except maternity leave)?

0. NO
1. YES

91. If the answer is yes, what was the reason? :

VII – WORKING CONDITIONS Now I am going to ask about your everyday working conditions

92. How do you collect waste?

1. Horse and cart
2. Wheelbarrow
3. Bicycle
4. On foot
5. Other

93. If other, please specify:

94. What type of material do you collect?

1. Paper/cardboard
2. Plastic
3. Iron
4. Electronic waste
5. Aluminum
6. Glass
7. Copper
8. Styrofoam
9. Other

95. of other, please specify:

96. Do you come into contact with decomposing (rotting) garbage?

0. NO
1. YES

97. When working, do you come into contact with any of the following animals? :

1. Dogs
2. Cats
3. Birds
4. Horses
5. Poisonous animals (spiders, scorpions)
6. Rodents (rats, guinea pigs)
7. Reptiles (snakes, lizards)
8. Organic material (disposable diapers, toilet paper)
9. Hospital waste (gauze, disposable syringes, needles)

98. In the last month, have you come into contact with:

1. Smoke
2. Batteries
3. Oil
4. Grease
5. Insecticides
6. Solvents
7. Paint
8. Cleaning products
9. Medicine
10. Aerosols
11. Other toxic products

99. If other, please specify:

100. When working, do you handle containers used for chemical products (e.g.: bleach, ajax, chemical disinfectants, caustic soda, acid)?

0. NO
1. YES

VIII – BIOMETRIC INFORMATION Now I am going to ask about your weight and height

101. How much do you currently weigh? :

102. What is your height?

in cm

IX – HEALTH STATUS Now I am going to ask about your health and any diseases you may have had

CHRONIC DISEASES Now I am going to ask about other health problems

103. When was the last time you had your blood pressure taken? :

1. Less than 6 months ago
2. 6 months to 1 year ago
3. 1 to 2 years ago
4. 2 to 3 years ago
5. 3 years ago or more
6. Never

104. Has a doctor ever diagnosed you with high blood pressure? (If the answer is no, go to question 107)

0. NO
1. YES

105. How old were you when you were first diagnosed with high blood pressure?

106. When was the last time you received medical treatment for high blood pressure? :

1. Less than 6 months ago
2. 6 months to 1 year ago
3. 1 to 2 years ago
4. 2 to 3 years ago
5. 3 years ago or more
6. Never

107. When was the last time you had a blood test to measure your blood sugar level?

1. Less than 6 months ago
2. 6 months to 1 year ago
3. 1 to 2 years ago
4. 2 to 3 years ago
5. 3 years ago or more
6. Never

108. Has a doctor ever diagnosed you with diabetes or high blood sugar? (If the answer is no, got to question 111)

0. NO
1. YES

109. How old were you when you were first diagnosed with diabetes?

110. When was the last time you received medical treatment for diabetes? :

1. Less than 6 months ago
2. 6 months to 1 year ago
3. 1 to 2 years ago
4. 2 to 3 years ago
5. 3 years ago or more
6. Never

111. When was the last time you had a blood test to measure your cholesterol and triglyceride levels? :

1. Less than 6 months ago
2. 6 months to 1 year ago
3. 1 to 2 years ago
4. 2 to 3 years ago
5. 3 years ago or more
6. Never

112. Has a doctor ever diagnosed you with high cholesterol?
(If the answer is no, go to question 114)

0. NO
1. YES

113. How old were you when you were first diagnosed with high cholesterol?

114. Has a doctor ever diagnosed you having a stroke?
(If the answer is no, go to question 116)

0. NO
1. YES

115. How old were you when you were first diagnosed having a stroke?

116. Has a doctor ever diagnosed you with chronic kidney disease?
(If the answer is no, go to question 117)

0. NO
1. YES

117. How old were you when you were first diagnosed with chronic kidney disease?

X – ENVIRONMENTAL HEALTH PROBLEMS Now I am going to ask some questions about your intestinal health

118. Have you had intestinal worms in the last month?

0. NO
1. YES

119. How do you know? :

1.Worms observed after evacuation

2.Stool test

3.Medical diagnosis

4.Other

120. Have you ever had leptospirosis?
(If the answer is no, go to question 123).

0. NO

1. YES

121. How long ago? :

122. How do you know?

1.Diagnosed by a doctor

2.Medical tests

3.Other

123. Have you had diarrhea in the last month?

0. NO

1. YES

124. When did it start?

125. Do you still have it?

0. NO

1. YES

126. Have you ever had dengue fever, Zika virus or chikungunha?

0. NO

1. YES

127. If yes, which one? :

1. Dengue fever
2. Zika virus
3. Chikungunha

128. Did you experience any related complications? :

129. Has a doctor ever diagnosed you with hepatitis A?

0. NO

1. YES

XI – MUSCULOSKELETAL PROBLEMS Now I am going to ask some questions about your physical health

130. In the last year, have you experienced any pain or discomfort in any of the following:

1. Neck
2. Shoulders
3. Elbows
4. Wrists or hands
5. Upper back
6. Lower back
7. Thighs
8. Legs
9. Knees
10. Ankles
11. Arms

131. Has this ever prevented you from doing household chores or other everyday tasks in the last year?

0. NO
1. YES

132. Have you experienced this pain in the last 7 days?

0. NO
1. YES

133. Has a doctor ever diagnosed you with arthritis or rheumatism?

0. NO
1. YES

134. Have you ever had any chronic spinal problems such as chronic back or neck pain, lower back pain, sciatica or spinal disk problems?

0. NO
1. YES

135. How old were you when you started experiencing back pain?

SKIN PROBLEMS Now I am going to ask you questions about your skin

136. Have you experienced any of the following in the last year:

1. Itching and rash
2. Sores with pus
3. Blisters
4. Calluses
5. Nail problems
6. Lice
7. Scabies
8. Sand flea bites
9. Myiasis, human botfly infection
10. Shingles
11. Marks on the skin accompanied by numbness

137. Others (please specify):

XIII – CARDIORESPIRATORY PROBLEMS Now I am going to ask some questions about your heart and lungs

138. Has a doctor ever diagnosed you with heart disease?

0. NO
1. YES

139. Which one(s)?

1. Heart attack
2. Heart failure
3. Angina

140. How old were you when you were first diagnosed with heart disease?

141. If you have been diagnosed with a different type of heart disease, please specify:

142. Has a doctor ever diagnosed you with bronchitis?

0. NO
1. YES

143. Has a doctor ever diagnosed you with asthma?

0. NO
1. YES

144. Have you had an asthma attack in the last 12 months?

0. NO
1. YES

145. Do you have or have you ever had any other type of lung problem?
(If the answer is no, go to question 148)

0. NO
1. YES

146. Please specify

147. How old were you when you were first diagnosed with a lung disease?

148. Has a doctor ever diagnosed you with tuberculosis?

0. NO
1. YES

149. Did you receive treatment?

0. NO
1. YES

150. Did you complete the treatment?

0. NO
1. YES

151. Have you had pneumonia in the last year?
(If the answer is no, go to question 156).

0. NO
1. YES

152. How many times? :

153. The last time you had it, who told you it was pneumonia? :

1. Doctor
2. Nurse
3. Pharmacist
4. Other

154. Did you receive treatment? :

0. NO
1. YES

155. Where? :

1. Home
2. Hospital
3. Healthcare Center
4. Outpatient Center
5. Pharmacy

XIV - CANCER Now I am going to ask you some questions about cancer

156. Have you ever been diagnosed with cancer?
(If the answer is no, go to question 162).

0. NO
1. YES

157. When first diagnosed, what type of cancer did you have?

1. Lung
2. Intestine
3. Stomach
4. Breast
5. Cervix (women only)

6. Prostate (men only)
7. Skin
8. Mouth
9. Other (specify) ____________________________

157.1 – If other, please specify:

158. How old were you when you were first diagnosed with cancer?

159. In general, to what extent did the cancer or related problems limit your everyday activities (such as working, doing household chores, etc.)?

1. Not at all
2. A little
3. Some
4. Quite a lot
5. A lot

160. Did you receive treatment?

0. NO
1. YES

161. Where?

1. Hospital
2. Outpatient care

162. What type of treatment?

1. Surgery
2. Chemotherapy
3. Radiotherapy

XV - MENTAL HEALTH Now I am going to ask some questions about your mental health

163. Do you often experience headaches?

0. NO
1. YES

164. Do you suffer from a lack of appetite?

0. NO
1. YES

165. Do you sleep poorly?

0. NO
1. YES

166. Do you get easily frightened?

0. NO
1. YES

167. Do you have hand tremors?

0. NO
1. YES

168. Do you feel nervous, tense or worried?

0. NO
1 YES

169. Do you have poor digestion?

0. NO
1. YES

171. Have you been feeling sad lately?

0. NO

1. YES

172. Have you been crying more than usual?

0. NO
1. YES

173. Do you get enjoyment from everyday activities?

0. NO
1. YES

174. Do you find it difficult to make decisions?

0. NO
1. YES

175. Do you feel your work is strenuous and causes you suffering?

0. NO
1. YES

176. Do you think your work plays an important role in your life?

0. NO
1. YES

177. Have you lost interest in things?

0. NO
1. YES

178. Do you feel worthless?

0. NO
1. YES

179. Have you ever thought about committing suicide?

0. NO
1. YES

180. Do you feel tired all the time?

0. NO
1. YES

181. Has a mental health professional (such as a psychiatrist or psychologist) ever diagnosed you with depression?
(If the answer is no, go to question 183).

0. NO
1. YES

182. How old were you when you were first diagnosed with depression?

183. Has a mental health professional (such as a psychiatrist or psychologist) ever diagnosed you with another type of mental disorder, such as schizophrenia, bipolar disorder, psychosis or obsessive compulsive disorder (OCD)?
(If the answer is no, go to question 187).

184. If yes, which one?

1. Schizophrenia
2. Bipolar disorder
3. OCD (Obsessive Compulsive Disorder)
4. Other

185. If other, please specify:

186. How old were you when you were first diagnosed with a mental disorder?

XVI – SEXUALLY TRANSMITTED DISEASES

187. Have you ever had hepatitis?

0. NO
1. YES

188. What type?

1. A
2. B
3. C
9. Don't know

189. Did you receive treatment?

0. NO
1. YES

190. Did you complete the treatment?

0. NO
1. YES

191. Have you ever had any other sexually transmitted diseases (STDs)?

0. NO
1. YES

192. If yes, please specify:

XVII - USE OF MEDICINES Now I am going to ask some questions about your use of medicines

193. Do you use any continuous medication? (Every day) :
(If the answer is no, go to question 196).

0. NO
1. YES

194. What problems do you use this medication for? :

1. High blood pressure
2. Angina? Chest pain or discomfort?
3. Diabetes or high blood sugar
4. Asthma
5. Hepatitis
6. Aids
7. High cholesterol or triglycerides
8. Birth control
9. Insomnia
10. Depression
11. Arthritis/arthrosis
12. Pain

194.1 Others (Specify):

195. Are you able to obtain all the medication you need? (either by buying or receiving free from health clinics)

0. NO
1. YES

196. Have you ever found medication in the trash?

0. NO
1. YES

197. Have you ever used medication you found in garbage?

0. NO
1. YES

198. Do you know anyone who has used medication found in garbage? :

0. NO
1. YES

XVIII – WOMEN'S HEALTH Now I am going to ask about your reproductive history.

199. Have you ever been pregnant? (Even if you never carried to term)?

0. NO
1. YES

200. How old were you at your first pregnancy?

201. How many times have you given birth?

202. How many of these were cesarean sections?

203. How many resulted in live births (that is, infants who showed signs of life at birth)?

204. Did any of your live born children subsequently die?

0. NO
1. YES

205. If yes, what was the cause? :

206. Have you ever suffered a miscarriage?

0. NO
1. YES

207. How many?

208. Have you ever had an abortion?

0. NO
1. YES

209. If yes, how many?

210. How many of your children were born with a health problem, disability or some type of malformation?

211. Blood pressure:

212. Remarks:

213. Have you ever had a Pap smear?

NO
YES

214. When was the last time you had a Pap smear? :

1. Less than 1 year ago
2. 1 to 2 years ago
3. 2 to 3 years ago
4. 3 or more years ago

XIX – ADDITIONAL SOCIOECONOMIC INFORMATION Now I am going to ask some questions about your schooling level

215. Can you read and write?

0. NO
1. YES

216. What was the highest level of schooling you completed?

0. None
1. Primary education (Elementary School)
2. Secondary education (High School)
3. Higher education (College)

217. What was the last grade you passed at school?

218. Is working as a waste picker your only source of income? :

0. NO
1. YES

219. Do you do other work to supplement your income as a waste picker? :

0. NO
1. YES

220. How much do you earn a month?

221. Do you receive:

1. A pension
2. Alimony/Child Support
3. Any other financial aid

221 - A – IF YOU RECEIVE OTHER FINACIAL AID, PLEASE SPECIFY

222. What is your monthly household income?

Save **Save**

Important: When saving, please wait for the confirmation screen. Your data will only be saved when the confirmation screen appears.

Click here if you have any questions about this form.

Page 1 of 1

Logotipo FormSus
